# Cerebrospinal fluid analysis in patients with COVID-19-associated central nervous system manifestations: a systematic review

**DOI:** 10.1590/0004-282X-ANP-2021-0117

**Published:** 2022-02-28

**Authors:** Renan Barros Domingues, Fernando Brunale Vilela de Moura Leite, Carlos Senne

**Affiliations:** 1 Senne Liquor Diagnóstico, São Paulo SP, Brazil. Senne Liquor Diagnóstico São Paulo SP Brazil

**Keywords:** COVID-19, Cerebrospinal Fluid, Encephalitis, Polymerase Chain Reaction, Headache, COVID-19, Líquido cefalorraquidiano, Encefalite, Reação em Cadeia da Polimerase, Cefaleia

## Abstract

**Background:**

Central nervous system (CNS) symptoms may occur in patients with acute COVID-19. The role of CSF examination in these patients remains to be established.

**Objective:**

A systematic review of CSF findings relating to COVID-19 was carried out.

**Methods:**

CSF parameters, including cytological and biochemical analyses, SARS-CoV-2 RT-PCR and other CSF markers, were recorded and analyzed among patients with acute COVID-19 and one of the following CNS syndromes: stroke, encephalopathy, encephalitis, inflammatory syndromes, seizure, headache and meningitis.

**Results:**

Increased white blood cells and/or increased protein concentration were found in 52.7% of the patients with encephalitis, 29.4% of the patients with encephalopathy and 46.7% of the patients with inflammatory syndromes (P < 0.05). CSF RT-PCR for SARS-CoV-2 was positive in 17.35% of the patients with encephalitis and less than 3.5% of the patients with encephalopathy or inflammatory syndromes (P < 0.05). Intrathecal production of immunoglobulins was found in only 8% of the cases. More than 85% of the patients had increased CSF cytokines and chemokines. Increased CSF neurofilament light chain (NfL) and CSF Tau were found in 71% and 36% of the cases, respectively.

**Conclusion:**

Non-specific inflammatory CSF abnormalities were frequently found in patients with COVID-19 CNS syndromes. The increase in neurodegeneration biomarkers suggests that neuronal damage occurs, with long-term consequences that are still unknown.

## RESUMO

**Antecedentes:**

O comprometimento do sistema nervoso central (SNC) pode ocorrer em pacientes com COVID-19. O papel do exame do LCR nesses casos ainda não foi precisamente estabelecido.

**Objetivo:**

Foi realizada uma revisão sistemática dos achados do LCR em pacientes com COVID-19 e comprometimento do SNC.

**Métodos:**

Os parâmetros do LCR, incluindo análises citológicas e bioquímicas, RT-PCR para o SARS-CoV-2, além de outros marcadores liquóricos, foram registrados e analisados ​​em pacientes com as seguintes síndromes: acidente vascular cerebral, encefalopatia, encefalite, síndromes inflamatórias, crises epilépticas, cefaleia e meningite.

**Resultados:**

Aumento de leucócitos e/ou aumento da concentração de proteína foram encontradas em 52,7% dos pacientes com encefalite, 29,4% dos pacientes com encefalopatia e 46,7% dos pacientes com outras síndromes inflamatórias (P<0,05). A RT-PCR em LCR ​​para identificação SARS-CoV-2 foi positiva em 17,35% dos pacientes com encefalite e menos de 3,5% dos pacientes com encefalopatia ou síndromes inflamatórias (P<0,05). A produção intratecal de imunoglobulinas foi encontrada em apenas 8% dos casos. Mais de 85% dos pacientes apresentavam aumento de citocinas e quimiocinas no LCR. Foi identificado aumento do neurofilamento de cadeia leve (NfL) em 71% e da proteína Tau em 36% dos casos.

**Conclusão:**

Alterações inflamatórias leves e inespecíficas foram frequentes em pacientes com manifestações neurológicas do SNC associadas à COVID-19. O aumento de biomarcadores de neurodegeneração sugere a possibilidade de dano neuronal, com consequências de longo prazo ainda desconhecidas.

## INTRODUCTION

The new coronavirus (SARS-CoV-2) appeared in Wuhan, China, and quickly evolved into a pandemic^1^. There have been reports of neurological manifestations associated with COVID-19. These manifestations include mild symptoms, such as headache, fatigue, hypogeusia and hyposmia; and severe manifestations, such as encephalitis, encephalopathy, Guillain-Barré syndrome (GBS) and stroke^2^.

Cerebrospinal fluid (CSF) analysis is essential in making diagnoses of infections of the central nervous system (CNS), since it can provide information about the inflammatory response and can identify the etiological agent^3^. However, the precision of CSF analysis among patients with acute COVID-19 and CNS manifestations has not yet been fully established^4^.

In this article, we present a systematic review of CSF findings among patients with COVID-19 and acute CNS symptoms and discuss the potential findings in different CNS syndromes. We also discuss the potential contribution of CSF analysis in understanding the underlying mechanisms in CNS manifestations associated with acute COVID-19.

## METHODS

MEDLINE (accessed from PubMed) was systematically searched from January 1, 2020, to April 30, 2021. The search strategy included the key words: “COVID 19” AND “Cerebrospinal Fluid”. Articles written in English, Spanish, Portuguese and French were included in this search. 

We included the following study types: case reports, descriptive retrospective studies and longitudinal studies. Review articles were not included. We only included studies that reported CSF findings. Studies reporting CSF findings from patients with COVID-19 and peripheral nervous system (PNS) manifestations were not included in the present analysis. We also excluded reports on multisystem inflammatory syndrome in children, associated with COVID-19. Cases in which an infectious etiology other than COVID-19 was identified to explain the neurological condition, either through conventional microbiology or through molecular methods, were also excluded. 

We retrieved the results from CSF analysis in the different clinical syndromes that were reported in the studies: stroke, encephalitis, encephalopathy, inflammatory syndromes, meningitis, seizures and headache. The clinical syndrome definitions used had some variation between studies but, in general, encephalopathy was defined as alteration of one or more brain functions that was attributed to systemic disease without structural brain lesions^4^, while encephalitis was characterized by typically focal neurological signs, with or without meningeal involvement. Neuroimaging studies and/or detection of inflammatory cells in the cerebrospinal fluid can identify meningeal involvement when it is present^4^^-^^14^. Other inflammatory syndromes considered included cases of acute disseminated encephalomyelitis (ADEM)^15^, neuromyelitis optica, clinically isolated syndrome (CIS)^7^ and acute hemorrhagic leukoencephalopathy^11^. When described in a study, the classification of epileptic seizures was reported. 

The following CSF parameters were sought and registered from the studies: opening CSF pressure; CSF white blood cells (WBC); CSF protein concentration; CSF SARS-CoV-2 RT-PCR; intrathecal immunoproduction (IgG index and/or CSF and serum oligoclonal bands [OCBs]); CSF specific antibodies (including anti-SARS-CoV-2, anti-myelin oligodendrocyte glycoprotein [anti-MOG], anti-aquaporin-4 [anti-AQ4], anti-N-methyl-D-aspartate [anti-NMDA], anti-glutamate decarboxylase [anti-GAD] and other autoimmune and paraneoplastic encephalitis antibodies; and also anti-cardiolipin); CSF interleukins (Ils); chemokines (CXCLs); tumor necrosis factor alpha (TNF-α); and neurodegeneration CSF biomarkers (ß-Amyloid, Tau, enolase, neurofilament light chain [NfL], glial fibrillary acid protein [GFAP] and α-synuclein). 

We statistically compared the CSF data from encephalitis, encephalopathy and inflammatory syndrome cases. The statistical comparisons were carried out using the chi-square test or Fisher’s exact test, depending on the number of observations in the contingency table. The significance level was set at P < 0.05. 

## RESULTS

A total of 222 articles were identified. Among these, we selected 75 articles in accordance with the search criteria. The study types were case report (37 studies), retrospective (38 studies) and longitudinal (one study). 

A total of 663 patients were included in these 75 studies. The clinical diagnoses of CNS syndromes among the patients reported in the studies were the following: hemorrhagic stroke (9 cases; 1.35%), ischemic stroke (16 cases; 2.41%), encephalitis (81 cases; 12.25%), encephalopathy (264 cases; 39.82%), headache (52 cases; 7.84%), other inflammatory syndromes (56 cases; 8.45%), meningitis (4 cases; 0.6%) and seizures (22 cases; 3.32%). The seizure types were described as motor (tonic-clonic) generalized onset seizures (2), focal non motor onset with impaired awareness (2) and unknown (13). The clinical syndrome was not defined by the authors of the studies in 159 cases. 

The findings of CSF parameters were as follows:

- Opening CSF pressure: this parameter was reported in 59 cases, which were 14 cases of headache, 13 cases of inflammatory syndromes, 30 cases of encephalitis and two cases of encephalopathy. Among these, 22% presented increased opening CSF pressure^5^^-^^9^. The results according to the different CNS clinical syndromes are shown in Table 1.

**Table 1.** Number of cases tested and number and percentage of cases with abnormal results from routine CSF analysis in different CNS syndromes.

Clinical syndrome	CSF pressure	CSF WBC	CSF protein	CSF RT-PCR
Ischemic stroke	0	0/4 (0%)	0/4 (0%)	0/3 (0%)
Encephalitis	2/30 (6.66%)	39/74 (52.70%)	25/54 (46.29%)	6/35 (17.14%)
Encephalopathy	1/2 (50%)	24/196 (12.24%)	42/153 (29.41%)	5/149 (3.35%)
Headache	6/14 (42.86%)	0/16 (0%)	1/16 (6.25%)	0/17 (0%)
Inflammatory syndromes	4/13 (32.5%)	18/48 (37.50%)	29/48 (41.66%)	1/31 (3.22%)
Meningitis	0	2/2 (100%)	1/1 (100%)	1/2 (50%)
Seizures	0	3/9 (33.34%)	4/5 (80%)	0/6 (0%)

CSF: cerebrospinal fluid; CSF pressure: elevated CSF open pressure; CSF WBC: increased CSF white blood cells; CSF protein: increased CSF protein concentration; CSF RT-PCR: detection of severe acute respiratory coronavirus 2 in CSF by means of reverse transcriptase polymerase chain reaction.

- CSF WBC: CSF WBC was reported in 349 cases. Increased CSF WBC was found in 86 cases (24.64%). Most cases with pleocytosis had less than 100 cells/mm^3^. In two cases of encephalitis there were more than 100 cells/mm^3^, in one case the CSF WBC was 115 cells/mm^3^ and in one case there was marked pleocytosis with 1920 cells/mm^3^. Overall, there was a predominance of lymphomononuclear cells^5^^-^^76^. The CSF WBC findings according to the CNS clinical syndromes are shown in Table 1.
- CSF protein concentration: This information was reported in 281 cases. Increased CSF protein concentration was found in 93 patients (33.09%). A mild increase in CSF protein concentration (< 100 mg/dl) was found in 269 patients, moderate increase (100-200 mg/dl) in nine cases and high increase (> 200 mg/dl) in three cases. The highest concentration was found in a patient with a CNS inflammatory syndrome with CSF protein concentration of 803 mg/dl^7^^,^^8^^,^^11^^-^^17^^,^^19^^,^^21^^,^^25^^-^^27^^,^^31^^,^^32^^,^^34^^-^^37^^,^^49^^-^^76^.
- SARS-CoV-2 CSF RT-PCR: A search for SARS-CoV-2 genetic material in CSF samples by means of RT-PCR was reported in 243 cases. Thirteen patients (5.34%) were found to be positive^5^^,^^8^^,^^10^^-^^12^^,^^14^^-^^25^^,^^27^^-^^29^^,^^32^^-^^46^^,^^49^^-^^77^. Among the cases classified as having encephalitis, the positivity rate was 17.14%. The percentages of positive RT-PCR results according to the clinical syndrome are shown in Table 1.
- Intrathecal immunoproduction and specific CSF antibodies: OCBs were found in 20% of the 138 patients on whom this test was performed. The most frequent pattern was type 4, with identical OCBs in CSF and serum, thus indicating systemic inflammation. The IgG index was negative in all 12 cases tested. Among the specific antibodies tested in CSF, anti-MOG was positive in one of two cases (50%), anti-NMDA was positive in three of 17 cases (11%) and anti-cardiolipin was positive in two of 11 cases (18%)^16^^,^^18^^,^^21^^,^^22^^,^^24^^,^^26^^,^^31^^,^^52^^,^^56^^,^^59^^,^^63^. The complete list of antibodies tested and their results are shown in Table 2.

**Table 2.** Number of cases tested and number of cases with abnormal results from the following CSF tests: intrathecal antibody synthesis tests, specific antibody identification, inflammation mediators such as interleukin and chemokine concentrations and neurodegeneration biomarkers in patients with acute COVID-19.

CSF test		Encephalopathy	Encephalitis	Inflammatory syndrome	IS	HS	Seizure	Headache	Not defined	Total	%
IgG and antibodies	OCB (total)	43	2	9	2	0	6	13	63	138	0
	OCB negative	17	0	7	2	0	3	13	57	99	72
	OCB type 2	2	0	0	0	0	0	0	6	8	8
	OCB type 4	23	0	2	0	0	3	0	0	28	20
	IgG index (total)	0	0	12	0	0	0	0	0	12	0
	IgG index (positive)	0	0	0	0	0	0	0	0	0	0
	ANTI-AQ-4 (total)	0	0	12	0	0	0	0	0	12	0
	ANTI-AQ-4 positive	0	0	0	0	0	0	0	0	0	0
	Anti-MOG (total)	0	0	2	0	0	0	0	0	2	0
	Anti-MOG (positive)	0	0	1	0	0	0	0	0	1	50
	IgG anti-SARS-CoV-2 (total)	14	2	0	3	2	0	0	0	10	0
	IgG anti-SARS-CoV-2 (positive)	6	0	0	1	1	0	0	0	0	38
	Anti-NMDA (total)	2	1	13	0	0	0	0	12	27	0
	Anti-NMDA (positive)	1	1	1	1	0	0	0	1	3	11
	Anti-GAD (total)	1	0	12	0	0	0	0	11	24	0
	Ant-GAD (positive)	0	0	0	0	0	0	0	0	0	0
	Other AI* (total)	1	0	0	0	0	0	0	11	12	0
	Other AI* (positive)	0	0	0	0	0	0	0	0	0	0
	Anti-cardiolipin (total)	0	0	0	0	0	0	0	11	11	0
	Anti-cardiolipin (positive)	0	0	0	0	0	0	0	2	2	18
Inflammatory markers	IL-6 (total)	3	39	2	12	5	3	0	58	122	0
	Increased Il-6	3	33	2	12	5	3	0	47	105	86
	Il-8 (total)	1	3	0	12	5	3	0	18	42	0
	Increased IL-8	1	3	0	12	5	3	0	18	42	100
	Il-10 (total)	1	3	2	0	0	0	0	0	6	0
	Increased Il-10	1	3	2	0	0	0	0	0	6	100
	TNF-alpha (total)	1	2	0	5	12	3	0	25	48	0
	Increased TNF-alpha	1	2	0	5	12	3	0	18	41	85
	MCP-1 (total)	0	1	0	0	0	0	0	0	1	0
	Increased MCP-1	0	1	0	0	0	0	0	0	1	100
	Il-2 (total)	0	22	2	0	0	0	0	0	24	0
	Increased Il-2	0	22	2	0	0	0	0	0	24	100
	Il-4 (total)	0	2	0	0	0	0	0	0	2	0
	Increased Il-4	0	2	0	0	0	0	0	0	2	100
	Il-12 (total)	0	2	0	0	0	0	0	7	9	0
	Increased Il-12	0	2	0	0	0	0	0	0	2	22
	Il-1ß (total)	0	0	0	0	0	0	0	18	18	0
	Increased Il-1ß	0	0	0	0	0	0	0	18	18	100%
	CXCL-2 (total)	0	22	0	0	0	0	0	0	22	0
	Increased CXCL-2	0	22	0	0	0	0	0	0	22	100%
	CXCL-8 (total)	0	0	2	0	0	0	0	0	2	0
	Increased CXCL-8	0	0	2	0	0	0	0	0	2	100%
	CXCL-10 (total)	1	3	2	0	0	0	0	0	6	0
	Increased CXCL-10	1	3	2	0	0	0	0	0	6	100%
CSF neurodegeneration biomarkers	ß-amyloid (total)	1	0	0	0	0	0	0	0	1	0
	Decreased ß-amyloid	0	0	0	0	0	0	0	0	0	0%
	Tau (total)	1	16	0	0	0	0	0	16	33	0
	Increased Tau	0	6	0	0	0	0	0	6	12	36%
	Enolase (total)	0	1	0	0	0	0	0	0	1	0
	Increased enolase	0	1	0	0	0	0	0	0	1	100%
	NfL (total)	0	17	0	0	0	0	0	34	51	0
	Increased NfL	0	8	0	0	0	0	0	28	36	71%
	GFAP (total)	0	17	0	0	0	0	0	16	33	0
	Increased GFAP	0	3	0	0	0	0	0	3	6	18%
	α-synuclein (total)	0	0	0	0	0	0	0	7	7	0
	Increased α-synuclein	0	0	0	0	0	0	0	0	0	0%

CSF: cerebrospinal fluid; IS: ischemic stroke; HS: hemorrhagic stroke; Total: number of cases tested; OCB: oligoclonal bands; AQ-4: aquaporin-4; MOG: myelin oligodendrocyte glycoprotein; NMDA: N-methyl-D-aspartate; GAD: glutamic acid decarboxylase; *Other AI: other CSF antibodies for autoimmune and paraneoplastic encephalitis; Il: interleukin; TNF-α: tumor necrosis factor alpha; MCP-1: monocyte chemoattractant protein -1; CXCL: chemokine; NfL: light neurofilament; GFAP: glial fibrillary acidic protein.

- Inflammatory mediators: The complete list of cytokines and chemokines tested and their results is shown in Table 2. The most frequently tested inflammatory mediators were Il-1ß, Il-2, Il-6, Il-8 and TNF-α. Increased CSF concentration of inflammatory mediators was found in at least 85% of the cases in which they were tested. The only exception was in relation to Il-12, which was increased in only 22% of the cases, although it had only been tested in 18 cases^8^^,^^12^^,^^13^^,^^36^^,^^47^^,^^48^^,^^60^^,^^67^^,^^70^^,^^7^ ^6^.
- Neurodegeneration CSF biomarkers: The most frequently tested CSF neurodegeneration biomarkers were Tau protein, NfL and GFAP, in 33, 51 and 33 cases, respectively. Increased levels were found in 36%, 71% and 18%, respectively^9^^,^^61^^,^^66^^,^^70^^,^^77^. One study found a correlation between Tau and NfL levels and the opening CSF pressure^9^. The complete list of biomarkers and their results is shown in Table 2.
- Statistical analyses: Table 3 shows the statistical comparison between CSF data from patients with encephalitis, encephalopathy and inflammatory syndromes. The percentage of increased WBC in patients with encephalitis was significantly higher than in cases with encephalopathy or inflammatory syndrome. Patients with encephalitis and inflammatory syndrome had significantly higher protein concentration in the CSF than those with encephalopathy. The percentage of RT-PCR positivity was significantly higher among cases with encephalitis than in patients with encephalopathy and inflammatory syndromes. There was no difference between the groups regarding the presence or the type of OCBs, or the frequency of increased Il-6.

**Table 3.** Statistical comparisons of the following variables: increased WBC/mm^3^, increased protein concentration, percentage detection of the SARS-CoV-2 genome, percentage of type 2 and type 4 OCBs and percentage of cases with increased CSF IL-6, among patients classified as having encephalitis, encephalopathy or inflammatory syndromes, using the chi-square test or Fisher's exact test.

	Increased CSF WBC/mm^3^	Increased CSF protein concentration	RT-PCR	OCB type 2	OCB type 4	IL-6
Encephalitis	39 of 74 (52.7%)	24 of 54 (42.29%)	6 of 35 (17.14%)	0 of 2 (0%)	0 of 2 (0%)	33 of 39 (84.61%)
Encephalopathy	24 of 172 (12.24%)	42 of 153 (29.41%)	5 of 144 (3.35%)	2 of 42 (4.65%)	23 of 43 (53.48%)	3 of 3 (100%)
Inflammatory syndrome	18 of 48 (37.5%)	29 of 48 (41.66%)	1 of 31 (3.22%)	0 of 9 (0%)	2 of 9 (22.22%)	2 of 2 (100%)
P	< 0.05	< 0.05	< 0.05	NS	NS	NS

WBC: white blood cells; SARS-CoV-2: severe acute respiratory coronavirus 2; CSF: cerebrospinal fluid; IL-6: interleukin 6; OCB: oligoclonal bands; RT-PCR: detection of the genome of SARS-CoV-2 in the CSF with reverse transcriptase polymerase chain reaction; Il: interleukin; NS: not significant.

## DISCUSSION

Mild inflammatory changes in conventional CSF analyses were frequently found in patients with central neurological manifestations associated with COVID-19, and were seen more frequently in cases of encephalitis. The SARS-CoV-2 genome was found in a small percentage of cases, more commonly among those with encephalitis than in those with encephalopathy or other inflammatory syndromes. Most patients, regardless of the clinical syndrome, presented increased CSF cytokines and chemokines. CSF neurodegeneration markers were increased in ​​several cases. These data suggest that CSF analysis is useful in the clinical evaluation of these patients and may contribute to better understanding of the CNS neurological manifestations associated with COVID-19. However, with the present data, it is not yet possible to define the precise mechanisms involved in neuronal injury and in generation of neurological symptoms. 

The number of patients with ischemic and hemorrhagic stroke on whom CSF analysis was done was too small to allow any definitive conclusion about the role of CSF in these conditions. There were no abnormalities in the conventional CSF analysis in cases of ischemic stroke. The levels of Il-6, IL-8 and TNF-α were increased in all cases of ischemic and hemorrhagic stroke that were tested. It is still uncertain whether the inflammatory process revealed by these CSF measurements has a role in the pathogenesis of vascular injury associated with these clinical syndromes^21^^,^^24^^,^^43^^,^^49^^,^^57^. 

 Most patients with encephalitis had mild pleocytosis with mild to moderate increases in CSF protein concentration. Only in 17.14% of the encephalitis cases was the SARS-CoV-2 genome detected in CSF. Previous studies have shown that SARS-CoV-2 can invade the CNS, via the olfactory nerve or through hematogenous spread, which thus explains why RT-PCR was positive in some cases^7^. However, the precise role of neuroinvasion and the presence of SARS-CoV-2 within the CNS in encephalitis cases are still unknown. Interleukins, chemokines and TNF-α were increased in encephalitis cases^68^. One potential explanation for this is that inflammation has a more frequent role than direct virus-mediated neuronal injury; however, this hypothesis still needs confirmation. Another possibility is that the CSF viral load is extremely low, thus reducing the positivity of CSF RT-PCR^9^. Future studies comparing cases with and without detection of the viral genome and correlating CSF data with neuroimaging findings may help to elucidate the pathophysiology of COVID-19 encephalitis and perhaps may contribute to a more refined definition of these cases according to these findings. 

Changes to the conventional CSF analysis were found in a minority of cases of encephalopathy. The viral genome was found in the CSF in a small percentage of these patients. It is possible that systemic inflammation is a determining factor in these cases; however, this cannot be definitively stated since systemic inflammation was not evaluated in the present study with regard to encephalitis^5^^,^^8^^,^^10^^-^^12^^,^^14^^-^^25^^,^^27^^-^^29^^,^^32^^-^^76^. Other factors, such as hypoxia, metabolic alterations and drugs with effect on the CNS, may also have contributed but were not separately analyzed in the studies included in this review. CSF abnormalities were reported; pleocytosis was found in 12% of the encephalopathy cases and 3.35% had positive CSF RT-PCR for SARS-CoV-2. It is not possible to rule out the possibility that at least some of these cases were actually encephalitis rather than encephalopathy cases. The lack of uniformity in case definition between studies may have contributed to such heterogeneity. Distinguishing between COVID-19 encephalitis and COVID-19-associated encephalopathy can be difficult and much other information besides CSF needs to be considered, such as the clinical picture, electrophysiological findings and the brain magnetic resonance imaging findings^4^. Future studies should establish more homogeneous criteria for distinguishing between these syndromes and for assessing possible differences in neurological prognosis between these two conditions.

Patients with other inflammatory syndromes presented with conventional CSF changes similar to what is normally seen in patients with ADEM due to other etiologies, i.e. normal CSF or CSF with mild inflammatory changes. The SARS-CoV-2 genome was found in only one case of CIS and was not found in cases of ADEM. ADEM cases can have transient OCBs in the CSF and not in the serum. In the present review, no cases with SARS-CoV-2-associated ADEM had intrathecal immunoproduction, but it is important to mention that only nine cases of inflammatory syndromes underwent this analysis. It is possible that an autoimmune disorder triggered by SARS-CoV-2 infection occurred in these cases. One case that was positive for anti-MOG antibodies and two cases with anti-cardiolipin antibodies suggest the possibility of an immunological cross-reaction^26^. Unfortunately, these cases were not evaluated for the presence of CSF anti-SARS-CoV-2 antibodies^56^, which could have contributed to better understanding of the pathogenesis of this inflammatory brain injury. With the available data, it is not possible to establish whether ADEM associated with COVID-19 presents neurological differences in comparison with ADEM associated with other etiologies. The meaning of anti-NMDA positivity in a few cases, including some with a clinical pattern compatible with limbic encephalitis, still needs to be better elucidated. One hypothesis is that SARS-CoV-2 may trigger not only ADEM but also, on rare occasions, autoimmune limbic encephalitis^62^^,^^63^.

 The number of cases of patients with seizures and meningitis was too small for an accurate analysis on CSF findings in these groups^16^^,^^31^^,^^33^. It is more likely that seizures represent a manifestation of encephalopathy or encephalitis and not a specific clinical syndrome in the context of acute SARS-CoV-2 infection. Almost half of the patients with headache had increased opening pressure without other CSF abnormalities^6^^,^^9^^,^^48^. The mechanism for this increased pressure is unknown. It is possible that COVID-19-associated coagulopathy explains this increased opening pressure, for example, through thrombosis and stenosis in the venous drainage system. An alternative explanation is that a dysfunction in CSF production and absorption may occur during COVID-19. Both hypotheses still need to be better evaluated in future studies.

Increased levels of neurodegeneration markers were found in more than 40% of the patients with encephalitis in whom these markers were tested. One study showed higher levels of NfL in patients with encephalitis than in patients with encephalopathy^71^. It is possible that NfL determination, along with other CSF, clinical and neuroimaging data, contributes to refining the assessment of these patients. Also, abnormalities in neurodegeneration markers not only may be important in acute COVID-19 but also may potentially contribute to assessing long-term cognitive symptoms in patients with post-COVID-19 syndrome. In fact, previous studies showed a correlation between some markers of neurodegeneration and neuroinflammatory response in patients with COVID-19 and the risk of cognitive abnormalities^77^. Future studies providing prospective evaluations on cognition and neurodegenerative markers among patients who recovered from the acute phase of this disease may contribute to better understanding of the long-term neurological consequences of this infection. 

This study had limitations that deserve to be mentioned. The most important concerns the definition of cases. The distinction between encephalopathy and encephalitis is often difficult to ascertain, as both conditions can lead to lowered consciousness. The presence of epileptic seizures can be part of the context of either encephalopathy or encephalitis, so it is difficult to assess this manifestation in isolation. Considering the non-uniformity of criteria for defining cases between the different studies, it is possible that this influenced the interpretation of CSF data in these different syndromes. Other limitations included the fact that the studies were retrospective or case reports. The extensive period of review, taking into account the period of occurrence of the pandemic, and the analysis of a wide CSF database are strengths of this study.

In conclusion, the present review shows that CSF analysis has a role in evaluation of COVID-19-associated CNS disorders. CSF may also contribute to better understanding of the relationship between SARS-CoV-2 infection and the CNS. Prospective studies including assessment of CSF inflammatory and neurodegeneration biomarkers may possibly contribute to better understanding of the determinants of the neurological prognosis after acute COVID-19.
